# An Overview of Seafood Allergens: Structure–Allergenicity Relationship and Allergenicity Elimination Processing Techniques

**DOI:** 10.3390/foods14132241

**Published:** 2025-06-25

**Authors:** Yang Yang, Yehao Zhang, Xinrong He, Fei Huan, Jinli Chen, Meng Liu, Siyang He, Shinong Gu, Guangming Liu

**Affiliations:** 1 College of Environment and Public Health, Xiamen Huaxia University, 288 Tianma Road, Xiamen 361024, China; yangy@hxxy.edu.cn (Y.Y.); z13235990801@163.com (Y.Z.);; 2 College of Ocean Food and Biological Engineering, Jimei University, 43 Yindou Road, Xiamen 361021, China; 3 College of Marine Biology, Xiamen Ocean Vocational College, Applied Technology Engineering Center of Fujian Provincial Higher Education for Marine Food Nutrition Safety and Advanced Processing, Xiamen 361102, China; 4 State Key Laboratory of Microbial Metabolism, School of Life Sciences and Biotechnology, Shanghai Jiao Tong University, Shanghai 200240, China

**Keywords:** allergenic protein, elimination processing, epitopes, protein structure, seafood allergy

## Abstract

Seafood (fish, crustacean, and mollusk) allergy represents a critical global health issue. Food processing offers a viable strategy for allergenicity mitigation and serves as a critical intervention for seafood allergy prevention. This paper reviews recent advances in seafood allergen research, with particular focus on molecular properties, epitopes, and structure–allergenicity relationships, which are foundations for designing processing technologies to mitigate allergenicity. Furthermore, an analysis of how various food processing techniques modulate allergen structures and epitopes, ultimately affecting their allergenicity, was conducted. Current World Health Organization (WHO)/International Union of Immunological Societies (IUIS) listings include 44 fish allergens and 60 shellfish allergens, with their characterization enabling targeted processing approaches for allergenicity elimination. Physical processing techniques, including thermal and non-thermal treatment, can dramatically influence the conformational and linear epitopes by altering or destroying the structure of an allergen. Chemistry-based processing techniques (enzymatic-catalyzed cross-linking and glycation), which induce covalent/non-covalent interactions between allergens and various modifiers, can effectively mask epitopes through molecular complexation. Biological processing attenuates allergenicity by inducing protein unfolding, polypeptide chain uncoiling, and enzymatic degradation. Nevertheless, the structure–activity relationship of seafood allergens remains insufficiently elucidated, despite its critical role in guiding processing technologies for allergenicity elimination and elucidating the fundamental mechanisms involved.

## 1. Introduction

Food allergy is an adverse reaction induced by ingestion of specific food and food products, where the immune system recognizes certain food proteins as an allergen and generates an immune response that causes various allergies [1]. Food allergy affects nearly 10% of adults and 8% of children, and the prevalence has increased worldwide in recent decades [2]. More than 160 foods are known to cause food allergies, while a rather short list of foods account for most of the more serious disease burden, namely peanut, tree nuts, wheat, sesame seeds, fish, shellfish (crustacean and mollusk), egg, and milk, among which fish and shellfish belong to seafood [3].

**Figure 1:**
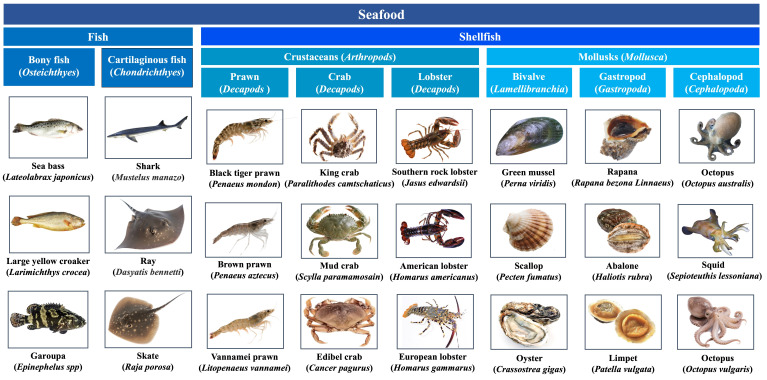
Classification of commonly consumed seafood species.

Seafood, also called “blue food”, is a general term for all edible aquatic organisms, of which fish and shellfish are mostly consumed (Figure 1) [4]. They are healthy delicacies with high protein content, healthy fats, vitamins, and minerals [5]. However, seafood allergy has become a serious global public health problem with increasing prevalence due to increasing consumption [6]. A recent study updated prevalence estimates for food allergy for the 2012–2021 period in Europe, showing the overall pooled estimates for fish and shellfish allergy of self-reported lifetime prevalence were 1.4% and 1.0%, respectively [7]. In certain parts of Europe, shellfish has become the leading food allergen [6]. In the USA, shellfish and fin fish ranked the most common allergenic foods, with an estimate of up to 2.9% and 0.9%, respectively. The prevalence rate of shellfish was even higher than that of peanut and tree nuts among adults [8].

Seafood allergy is of particular importance in the Asian region, with up to 7.7% prevalence in some Asian countries [9]. IgE sensitization to shellfish was as high as 10.6% among Singaporean children and up to 13.1% among the Chinese population, and 10.3% of Indian populations have shrimp specific IgE that exceeds 0.70 kU_A_/L [10]. Shellfish is also the leading cause of food allergy in China. The shellfish prevalence is 7.3% among Taiwanese, and over 2.0% of the Hong Kong population was positive for shrimp allergy via the skin prick test [6,10]. An epidemiological survey of self-reported food allergy performed by Feng et al. showed that shrimp was the main allergenic food among university students, followed by shellfish [11]. Fish allergy is less common in Asia than shellfish allergy, but its prevalence is still considerably high, affecting ~1.6% of the Vietnamese population and ~4.1% of the Singaporean population [12].

Unlike most other food allergies, seafood allergy is thought to persist for life in up to 90% of patients [13,14]. The avoidance of seafood-containing foods is the most effective measure to prevent seafood allergies. While this might result in various nutritional deficiency, there is an urgent need to seek effective processing to reduce seafood allergenicity. The presence of allergens contributes to seafood allergy; therefore, processing methods have been explored that aim at modifying allergens. In this review, we summarized current knowledge with respect to seafood allergens and various processing methods to decrease allergenicity. It is conceivable that the information will provide rationales for novel strategies to prevent and manage allergies to seafood.

## 2. Seafood Allergens and Their Molecular Properties

During the past decades, several allergenic proteins and related isoforms have been identified in commonly consumed seafood species and recorded in the World Health Organization and International Union of Immunological Societies (WHO/IUIS) (http://www.allergen.org, accessed on 1 May 2025). This section provides an overview of currently identified fish and shellfish allergens.

### 2.1. Fish Allergens

According to WHO/IUIS records as of May 2025, 44 distinct allergens originating from 22 species of fish have been officially characterized and classified, representing 13 kinds of proteins with confirmed allergenicity. Allergy to fish fillet is the most prevalent, but allergic reactions have also been reported to fish roe, fish gelatin, and fish blood (Table 1). Parvalbumin is the primary fish fillet allergen, followed by β-enolase and aldolase A. In recent years, tropomyosin and triosephosphate isomerase have been identified as novel fish allergens [15,16]. In addition, several minor fish allergens—such as creatine kinase, pyruvate kinase, lactate dehydrogenase, and glucose-6-phosphate isomerase—were also included in the WHO/IUIS list, and a characterization of their biochemical properties from a food allergen perspective is summarized in Table 2.

**Figure 2:**
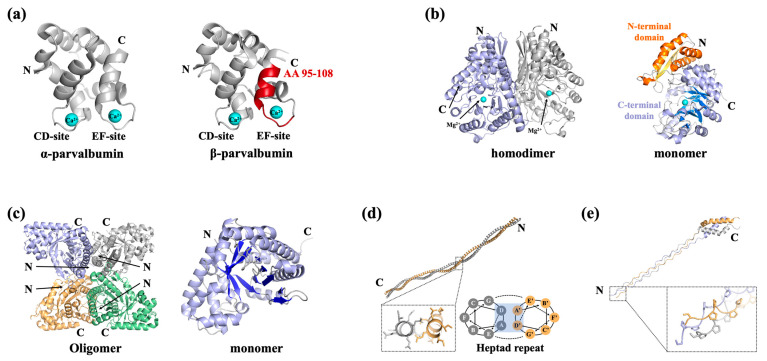
Representative structures of fish allergens. (**a**) Structure of α-parvalbumin (protein data bank, PDB: 5ZGM) and β-parvalbumin (5ZH6). The protein contains two Ca^2+^-binding domains, which are shown as cyan-colored balls; the important IgE-binding epitope of β-parvalbumin (AA 95–108) is colored red. (**b**) Structure of β-enolase (PDB: 2XSX). The β-enolase homodimer is composed of two identical monomers, represented in light blue and light grey for clarity. The monomer of β-enolase is comprised of the N-terminal domain (the α-helixes are represented in orange and the β-sheet is represented in yellow) and the C-terminal α/β-barrel (the α-helixes are represented in light blue and the β-sheet is represented in marine blue). A bound Mg^2+^ in the α/β-barrel is shown as a cyan-colored ball. (**c**) Structure of aldolase A (PDB: 4TU1). The aldolase A oligomer is composed of four identical monomers, represented in light blue, light grey, wheat, and pale green for clarity. The monomer of aldolase A folds into an α/β-barrel with 10 anti-parallel β-sheets (represented in marine blue) bundled by 13 α-helices (represent in light blue). (**d**) Structure of tropomyosin (PDB: 1C1G). The homodimer of tropomyosin is composed of two identically coiled subunits, represented in wheat and light grey for clarity. (**e**) Structure of collagen I (PDB: 5CVB). The collagen triple helix is formed via the association of three identical or different polypeptide chains, represented in wheat, light blue, and light grey for clarity. All structural information related to fish allergens was sourced from the RCSB Protein Data Bank (https://www.rcsb.org/).

The most characterized allergenic fish parvalbumins were demonstrated to be responsible for over 70% of allergic reactions to fish and fish products [17]. Most individuals with fish allergies exhibit allergic reactions to multiple fish species due to the high cross-reactivity of parvalbumins, which share 88–100% sequence similarity with conserved regions [18]. Parvalbumin is an extremely thermostable allergen in fish muscles with a molecular weight of 10~13 kDa. Parvalbumin can be divided into α-type and β-type, with the allergenicity of α-parvalbumin generally considered to be lower than β-parvalbumin [19]. Parvalbumin is a member of the EF hand-containing Ca^2+^-binding protein superfamily. Fish parvalbumins display a conserved spatial structure which consists of six α-helices, organized by the AB domain at the N-terminal and CD-EF domain at the C-terminal (Figure 2a) [20]. Two Ca^2+^-binding sites in the CD and EF domains can be found in the structure of parvalbumin, which are involved in its structural preservation. IgE-binding epitopes of various fish parvalbumins exist in a stereoscopic conformation maintained by Ca^2+^ binding; hence, depletion of Ca^2+^ leads to decreased IgE-binding capacity [19]. The epitopes of fish parvalbumins have been well documented, with most of the IgE-binding epitopes located on the loop regions between α-helices [19,21]. Perez-Cordo et al. found that amino acid (AA) 95~108 was recognized by all fish-allergic patients and can serve as a severity marker of fish allergy [21]. In a recent study by Huang et al., this region was also identified as an important IgE-binding epitope in β-parvalbumin [19]. It is worth noting that a three-dimensional structure shows this IgE-binding epitope located on the Ca^2+^-binding domain (Figure 2a, colored red).

Enolase is a glycolytic enzyme that catalyzes the reversible dehydration of 2-phosphoglycerate to phosphoenolpyruvate. In vertebrates, three enolase isotypes (α, β, and γ) have been identified, among which the β-isotype has been recognized as a fish allergen [22]. Kuehn et al. first characterized the thermolabile β-enolase as a novel allergen in three fish species, *Gadus morhua*, *Thunnus albacares*, and *Salmo salar*, demonstrating its reactivity in over 50% of fish-allergic patients [23]. Meanwhile, in a recent study by Wai et al., IgE reactivity to β-enolase was observed in 17.8% of tested subjects [24]. There is increasing evidence that β-enolase may play an important role in allergic disease, since it can sensitize individuals orally or via the respiratory tract, and even via skin, and the sequence conservation supports its cross-reactivity [22]. β-Enolase forms homodimers in muscle, and each monomer consists of two domains: the N-terminal domain folding into a two-layer sandwich composed of an anti-parallel β-sheet and an α-helical bundle; and a C-terminal domain folding into an α/β-barrel (Figure 2b). In addition, Mg^2+^ can be found in the α/β-barrel in each monomer [25]. However, the epitope of β-enolase remains to be characterized.

Aldolase A, another essential glycolytic enzyme with a molecular weight of 40 kDa, was first identified as an allergen in *Pacific salmon* in 2009 [26]. There are now four aldolase A enzymes registered as fish allergens in the WHO/IUIS, such as enolase, which is labile to thermal treatment and which can only be detected in raw fish extracts [23,27]. The structures of fish aldolase are yet to be determined. According to structures of other species, aldolase A seems to be present as an oligomeric protein, with each monomer folding into an α/β-barrel with 10 anti-parallel β-sheets bundled by 13 α-helices (Figure 2c) [28]. Although aldolase A has been shown to be heat-labile, its structure–allergenicity relationship remains unclear due to insufficient structural characterization and epitope mapping.

Vertebrate tropomyosin was reported to be a nonallergenic protein until recent studies challenged this belief. The tropomyosin from *Oreochromis mossambicus* was the first vertebrate tropomyosin identified as an allergen by immunological analyses [29]. In 2020, Ruethers et al. expanded the allergen repertoire of *Salmo salar* and *Pangasianodon hypophthalmus*, including a tropomyosin from *S. salar* and two tropomyosin isoforms from *P. hypophthalmus*; thus, these tropomyosins were registered with the WHO/IUIS [16]. In recent research by Li et al., tropomyosin was also confirmed as an important heat-stable allergen in *Lateolabrax japonicus* [30]. Tropomyosin has a molecular mass of around 35~38 kDa. It belongs to the actin-binding protein family, with a conserved spatial structure that is composed of two identically coiled subunits. Each of the tropomyosin subunits follows a repetitive “ABCDEF” heptamer pattern which contributes to the structural stability of the subunit (Figure 2d) [31,32]. The exceptionally stable α-helical coiled-coil secondary structure makes tropomyosin resistant to food processing, retaining high allergenic potential after heating and exposure to acidic conditions [16]. While Xu et al. revealed distinct digestive patterns between fish and shellfish tropomyosin, their study demonstrated that shrimp TM digestion products contained significantly more peptides matching known T/B cell epitopes compared to fish tropomyosin. This finding provides a plausible explanation for why tropomyosin serves as a major allergen in shellfish but only a minor allergen in fish species [33].

α-collagen was identified as a fish allergen in Japan in the early 2000s [34]. At present, α-collagens from *Lates calcarifer* and *S. salar* are registered with the WHO/IUIS as Lat c 6 and Sal s 6, respectively. Subsequent research has shown α-collagen to be a major fish allergen that could induce cross-reactivity [35]. Though limited cases of collagen-induced fish allergy have been reported in the past decades, Shimojo et al. found that IgE reactivity to fish collagen was associated with symptom severity [36]. Different genes code for distinct collagen chains, but all collagens share a conserved structure called a collagen triple helix, which is formed by the association of three identical or different polypeptide chains, with each chain displaying a conspicuous -Gly-X-Y- repetitive sequence (Figure 2e) [37,38]. Similar to tropomyosin, the triple-helical structure of collagen confers remarkable thermal stability. However, when subjected to heat treatment, collagen undergoes partial denaturation and becomes water soluble, thereby retaining its potential to induce anaphylactic reactions [39]. Due to both the underreporting of collagen allergy cases and insufficient structural characterization, comprehensive analysis of the structure–allergenicity relationship in collagen remains limited.

In addition to allergens mentioned above, other minor allergens have been identified in fish, most of which are enzymes. The cytosolic protein pyruvate kinase was first observed in a case in which a child appeared to have allergic symptoms after ingesting swordfish. Pyruvate kinase was recognized by the presence of IgE in patient serum, together with enolase, aldolase, and triosephosphate isomerase [40]. Triosephosphate isomerase, a key enzyme involved in glycolysis, is a known allergen from crustaceans and mollusks [41], and has also been recognized as a fish allergen in the research of Ruethers et al. [16]. The conserved structure, primarily composed of a (β/α)8-barrel motif prototype, was believed to contribute to its cross-reactivity [41]. The first case of allergy to creatine kinase was reported by Larco-Rojas et al. [42], and the allergenicity and cross-reactivity of creatine kinase has subsequently been confirmed by Ruethers et al. and Yang et al. [16,43]. Additionally, lactate dehydrogenase, glucose-6-phosphate isomerase, and glyceraldehyde-3-phosphate dehydrogenase were identified as fish allergens. Allergens can also be found in fish roe, including lipovitellin and β’-component, which are sub-fragments of vitellogenin [16,44]. Detailed information on their molecular properties and prevalence rate is to be further uncovered.

### 2.2. Shellfish Allergens

**Figure 3:**
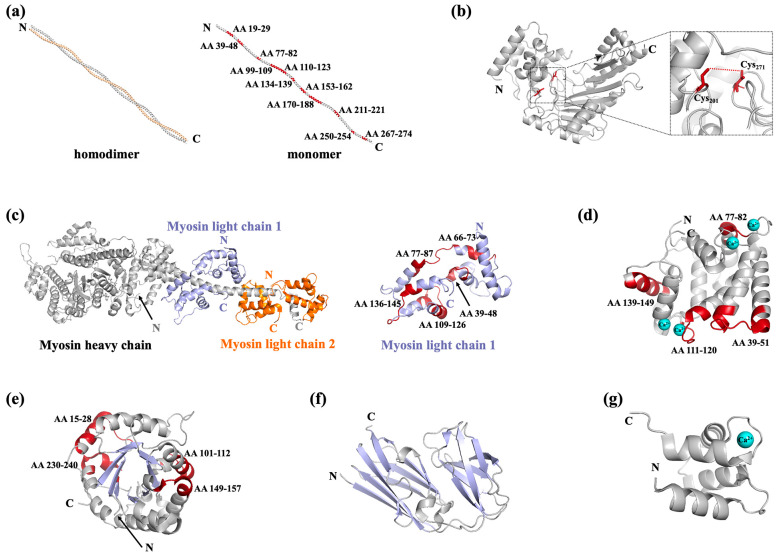
Representative structures of shellfish allergens. (**a**) Structure of tropomyosin (PDB: 1C1G), with the heat/digested stable epitopes of tropomyosin colored red. (**b**) Structure of arginine kinase (PDB: 5ZHQ), with the cysteines involved in the formation of disulfide bonds colored red and the intramolecular disulfide bond indicated by a red dashed line. (**c**) Structure of myosins (PDB: 3I5F). Myosin is composed of a heavy chain and two light chains, represented in light grey, light blue, and orange, respectively. The heat/digested stable epitopes of myosin light chain 2 are colored red. (**d**) Structure of sarcoplasmic calcium binding protein (PDB: 7WBO), with four bound Ca^2+^ shown as cyan-colored balls and the heat/digested stable epitopes of sarcoplasmic calcium binding protein colored red. (**e**) Structure of triosephosphate isomerase (PDB: 5EYW), with its eight central β-strands colored light blue for clarity and its heat/digested stable epitopes colored red. (**f**) Structure of filamin C (partial, PDB: 7VZO), with the β-sheets colored light blue. (**g**) Structure of troponin C, with a bound Ca^2+^ shown as a cyan-colored ball. All structural information related to shellfish allergens was sourced from the RCSB Protein Data Bank (https://www.rcsb.org/).

Hoffman et al. identified the IgE-binding tropomyosin in *Penaeus aztecus* for the first time in 1981 [45]. Since then, tropomyosin has been identified in various species and has subsequently been considered to be the major allergen in shellfish responsible for the cross-reactivity of many invertebrate species [46,47]. As showed in Table 1, Table 2 and Table 3, it is an allergen found in all three types of seafood, in fish, crustaceans, and mollusks. The α-helical coiled-coil dimeric structure is highly conserved across invertebrates and vertebrates, while vertebrate tropomyosin shows closer helix–helix packing of alanine clusters in which more enzymatic cleavage sites are accessible, leading to significantly weaker resistance to simulated gastric digestion than invertebrate tropomyosin [48]. Liu et al. found 11 heat/digested stable linear epitopes of tropomyosin, which are considered to be the primary cause of allergenicity (Table 2, Figure 3a) [49]. In the original research on tropomyosin, linear epitopes were thought to be the priority in tropomyosin because of its structural features, while some recent studies have revealed the existence and importance of its conformational epitopes [50,51]. Despite the limited structural data on conformational epitopes, current evidence suggests that both linear and conformational epitopes of tropomyosin appear to be evenly distributed across the protein.

Since Yu et al. identified arginine kinase as a novel shrimp allergen, it has subsequently been recognized as an allergen in many other kinds of shellfish [17,52]. Arginine kinase in seven crustaceans and one mollusk has now been officially recognized by the WHO/IUIS as a food allergen and has proved to be responsible for cross-reaction among invertebrates [53] (Table 3). Arginine kinase and creatine kinase are members of the phosphagen kinase family, with the conserved spatial structure of a canonical fold composed of an α-helical N-terminal domain and an α-β C-terminal domain (Figure 3b) [53]. This structure is easily disrupted by the breakage of the intramolecular disulfide bond between Cys_201_ and Cys_271_ [54]. In addition, arginine kinase is a thermal-sensitive protein which forms multimers in temperatures higher than 46 °C [55]. In arginine kinase, the conformational IgE epitopes are more predominant, providing a reason for the decreasing IgE-binding activity of denatured arginine kinase [54,56]. Heat/digested stable linear epitopes were also found in arginine kinase, which are crucial for its capacity to induce anaphylaxis (Table 2) [49].

Paramyosin was initially identified as a major respiratory allergen, and in the past decade, it has been identified as an allergen in mollusks. Yu et al. identified paramyosin as a novel allergen in *Rapana venosa*, which has been recognized by the WHO/IUIS as Rap v 2 [57] (Table 3). This allergen has proven to be a risk factor in mollusc–mite cross-reactivity [58]. Paramyosin belongs to the actin-binding protein family and is sometimes referred to as “water-insoluble tropomyosin” or “tropomyosin-A” because of its conserved α-helical secondary structure. It is believed that the α-helical secondary structure folds into a globular spatial structure, which is different from the long chain structure of tropomyosin [59]. Moreover, paramyosin is not as stable as tropomyosin with respect to heating treatment, which probably results from its structural property (Table 2). Ca^2+^-binding sites also exist in the sequence of paramyosin (Table 2), while the structure–allergenicity relationship remains to be explored.

Myosins are important functional muscle proteins belonging to the actin-binding family. They are multimeric proteins, with each subunit composed of a heavy chain with a molecular mass of ~200 kDa and two light chains with a molecular mass of ~18–20 kDa (Table 2, Figure 3c) [60]. The heavy chain is composed of a globular motor domain and a coiled-coil structure tail (Figure 3c, colored light grey); two light chains wrap around the neck region of each myosin heavy chain (Figure 3c, with myosin light chain 1 and 2 colored light blue and orange, respectively). The myosin heavy chain has been recognized as an allergen in fish and shrimp, and is also involved in the cross-reactivity between crustacean and mite [61,62,63]. Moreover, both myosin light chain 1 and myosin light chain 2 have been reported as food allergens. Ayuso et al. identified myosin light chain 2 as a major allergen of *Litopenaeus vannamei* (Lit v 3) that was recognized by 55% of shrimp-allergic subjects, obtaining a systematic name in the WHO/IUIS list [64] (Table 2). Subsequently, myosin light chain 2 from *Homarus americanus* (Hom a 3) and *Penaeus monodon* (Pen m 3) have also been recognized as allergens [65]. Additionally, myosin light chain 1 from *Artemia franciscana* (Art f 5), *Crangon crangon* (Cra c 5), *Scylla paramamosain* (Scy p 3), and *Procambarus clarkii* (Pro c 5) have also been identified as allergens in crustaceans [66,67,68]. The myosin light chains are mainly composed of α-helixes and random coils and show a canonical lobe with an N-terminal domain and a C-terminal domain (Figure 3c), with the epitopes distributed in the α-helixes with a highly conserved sequence [67,68]. Though Ca^2+^-binding sites can be found in the structures of myosin light chains, the influence of bound Ca^2+^ with respect to its allergenicity remains to be revealed. Myosin light chain 1 was found to be resistant to thermal treatment and extreme pH [69]. The digestion product of myosin light chain 1 contains 5 IgE-reactive epitopes (Table 2, Figure 3c, colored red) [49], while information on the physicochemical properties of myosin light chain 2 is lacking.

Sarcoplasmic calcium-binding protein was initially identified as a minor allergen in saltwater shrimp [70]. In recent years, the allergenicity and cross-reactivity of sarcoplasmic calcium-binding protein has further been demonstrated at the molecular level in other crustaceans and mollusks (Table 2 and Table 3) [71,72]. Sarcoplasmic calcium-binding protein belongs to the calcium-binding family. There are four EF-hand motifs in its structure, two or three of which individually contain a Ca^2+^-binding site corresponding to the loop region of 12 amino acid residues (Figure 3d) [73]. Sarcoplasmic calcium-binding protein is a heat-resistant allergen. Liu et al. identified four heat/digested peptides (AA 39–51, 77–82, 111–120, 139–149) that are crucial for its allergenicity (Table 2, Figure 3d, colored red) [49]; of note, two of these peptides are located in the Ca^2+^-binding sites (Figure 3d). Additionally, several linear and conformational epitopes are also located in the Ca^2+^-binding sites [71,73]. Despite the heat-resistance of sarcoplasmic calcium-binding protein, depletion of its Ca^2+^ leads to obvious conformation variation, which consequently reduces its immunoreactivity [71]. It can be speculated that Ca^2+^ depletion might influence IgE reactivity by either changing the accessible surface of the IgE recognition region or disrupting the structure for conformational epitope formation.

Triosephosphate isomerase in *C. crangon* (Cra c 8) was the first identified allergenic crustacean triosephosphate isomerase, having approximately 20% sensitization frequency [66]. The three-dimensional structure of triosephosphate isomerase is primarily composed of a (β/α)_8_-barrel motif prototype formed by eight central β-strands surrounded by eight α-helices joined by loops [41]. This structure is easily disrupted by heat treatment, accompanied with a reduction in IgE-binding activity [74]. Four heat/digested peptides were found to be distributed in either surface α-helices or intramolecular β-strands (Table 2, Figure 3e, colored red); meanwhile, there are two conformational epitope regions located on the protruding surface areas [41,49,75]. Filamin C first received notice for its cross-reactivity with triosephosphate isomerase in *P. clarkii* [74], and now it has also been identified as an allergen in *S. paramamosain* [76]. Different from other actin-binding family members, filamin C has been found to be thermal sensitive, and its decreasing IgE-binding capacity can be observed in cases of extreme pH [74]. The digested product of filamin C retains 50% IgE-binding activity [74], indicating the existence of digested resistant peptides. The overall spatial structure of filamin C is not available at present; nevertheless, He et al. determined the crystal structure of its allergic predominant region (AA 336–531), which displays a barrel structure composed of 16 β-strands and two α-helices (Figure 3f). A conformational epitope and six linear epitopes were found to be located in this region [77].

Other shellfish allergens are also identified, such as troponin C/I, hemocyanin, fatty acid-binding protein, ovary development-related protein, and glycogen phosphorylase-like protein (Table 3). Troponin is composed of three subunits, suffixed C, I, and T, with troponin C and I being registered as crustacean allergens [78]. Like other members of the actin-binding protein family, troponin C is mainly composed of α-helixes and random coils; in addition, EF-hand motifs, which contain functional Ca^2+^-binding loops, can be found in the structure (Figure 3g) [79]. There is no evidence of the influence of Ca^2+^ binding on its allergenicity. Allergenic hemocyanin can be found in shrimp cephalothorax or crab roe, and it has been recommended as a tool for shrimp and crab allergy diagnostics that distinguish it from allergies to muscle [80,81]. Hemocyanin is also an inhaled allergen which is reported to induce cross-reactivity between crustaceans and insects [80,82]. Ovary development-related protein has been registered with the WHO/IUIS as a crab allergen (Eri s 2); however, neither physicochemical and digestion properties nor epitope and structural information were reported [83]. Recently, Múnera et al. identified the 15 kDa fatty acid-binding protein as a novel shrimp allergen, naming it Lit v 13. Fatty acid-binding protein, together with the house dust mite allergen Der p 13, belong to the lipid-binding protein superfamily. Cross-reactivity can be observed between Lit v 13 and Der p 13, with the highly conserved AA 54–72 as the most involved region in the cross-reactivity [84]. In addition, Wai et al. identified glycogen phosphorylase-like protein in *P. monodon* (Pen m 14) as a novel allergen in addition to the fatty acid-binding protein (Pen m 13) [85].

The above information consolidates the documented seafood allergens to date and provides an analysis of their molecular structures, immunodominant epitopes, and key physicochemical properties. Analyses have demonstrated that the majority of characterized seafood allergens are functionally restricted to three principal protein categories: calcium-binding proteins, actin-binding proteins, and enzymatic proteins, with the first two categories exhibiting superior heat resistance (Table 2). Notably, allergens within each category exhibit similar physicochemical properties. Allergenic calcium-binding proteins, including parvalbumins and sarcoplasmic calcium-binding protein, preserve evolutionarily conserved EF-hand domains that form the structural basis for Ca^2+^ chelation and likely contribute to IgE reactivity. Actin-binding proteins, particularly tropomyosin and paramyosin, maintain highly conserved coiled-coil α-helical domains. These structurally stable motifs, characterized by their seven-residue (heptad) repeat pattern (Figure 2), confer remarkable thermal stability, enabling these allergens to maintain their immunoreactivity even after extensive heat processing. Enzymatic protein allergens, including enolase, aldolase, and arginine kinase, demonstrate marked thermolability, typically undergoing structural denaturation and loss of IgE-binding capacity during thermal treatment. The allergen categories presented in this study guide food processors in matching treatment methods to specific protein characteristics. Moreover, mapped epitope data supports the design of processing strategies that specifically modify immunodominant regions.

## 3. Effect of Processing Techniques on Seafood Allergens

Though avoidance of seafood and seafood products is the basic and common measure in managing seafood allergies, a long-term strict seafood-free diet might lead to a dietary imbalance. Therefore, food processing techniques that reduce or eliminate the allergenicity of seafood have attracted research studies. There are many reports about processing based on physics, chemistry, and biology techniques, or on a combination of different techniques. In general, processing may destroy existing epitopes of an allergen or may generate new ones because of changes involving protein conformation. In some circumstances, the structural changing of an allergen during processing is accompanied with more sites that are accessible to proteolytic enzymes, which produce hypoallergenic hydrolysates unable to elicit immune responses. This section reviews current knowledge with respect to how food processing modifies the allergenic potential of seafood.

### 3.1. Physical Processing Techniques

Physical processing techniques include thermal treatments (e.g., boiling, steaming, baking, frying, microwave) and non-thermal treatments (e.g., high-pressure processing, ultrasound, irradiation, cold plasma). For seafood processing, traditional heating methods (boiling, steaming, frying, etc.) and ultrasound are commonly applied treatments.

#### 3.1.1. Thermal Treatments

**Figure 4:**
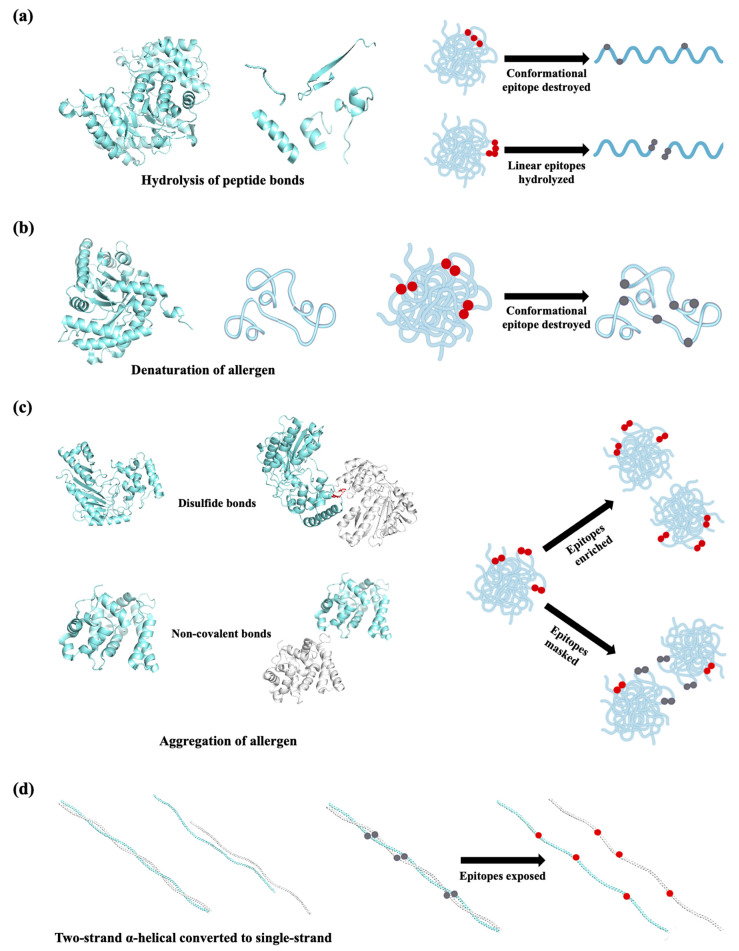
Schematic representation of thermal processing methods with respect to structure/epitope changes to seafood allergens. (**a**) Peptide bond hydrolysis during thermal treatment; (**b**) heating-induced denaturation of allergen; (**c**) aggregation of allergens during heating; (**d**) the two-strand α-helical coiled-coil structure converted to a single-stranded α-helix during thermal processing. The epitopes are indicated with red dots and the destroyed/masked ones are shown in grey. All structural information related to allergens was sourced from the RCSB Protein Data Bank (PDB; https://www.rcsb.org/).

The effect of thermal processing on food allergens is of major interest for allergic patients who need to avoid all food containing active allergens. Thermal treatments of food proteins induce modifications, including hydrolysis of peptide bonds, aggregation by disulfide and non-covalent bonds, denaturation, and reactions with other food components, such as carbohydrates and lipids [86]. For seafood allergens, the degradation, molecular aggregation, and denaturation of proteins are the primary factors altering allergenicity (Figure 4). The effect of thermal processing on fish allergenicity has been shown to be species and allergen dependent. Fish allergens such as aldolase, enolase, and creatine kinase have been described as thermolabile (Figure 4a). Enolases from *Gadus morhua* and *Thunnus albacore* lost their IgE-binding activity after heating at 90 °C for 1 min, a treatment condition that can sharply reduce the IgE-binding activity of aldolases at the same time [23]. In addition, creatine kinase from *Thunnus tonggol* was found to be undetected after boiling for 15 min [87]. Kubota et al. focused on the allergenicity of *Pacific mackerel* parvalbumin influenced by heat treatment. They found a more than 50% reduction in allergenicity by heating at 80 °C for 10 min and a complete loss of IgE reactivity to parvalbumin heated at 140 °C [88]. By detecting the binding activity of heated parvalbumin to the monoclonal antibody PARV-19, which recognizes parvalbumin in a Ca^2+^-dependent manner, the authors revealed the mechanism of allergenicity decreasing from conformational changes owing to Ca^2+^ depletion with the heat [88]. A recent investigation by Liang et al. also demonstrated that parvalbumin from *Scombriformes*, *Scorpaeniformes*, and *Tetraodontiformes* species showed weak or no immunoreactivity as a result of losing the Ca^2+^-dependent epitope [89]. However, in some species, such as *Cyprinus carpio* and the order of *Perciformes*, parvalbumin showed increased immunoreactivity to antibodies, due to an enrichment of parvalbumin upon aggregation during heat treatment [90].

Heat treatment appears to exert a low impact on crustacean allergens because most of them are thermostable, except for triosephosphate isomerase, filamin C, and arginine kinase. The IgE-binding activity of triosephosphate isomerase was reduced by increasing the temperature higher than 60 °C, accompanied by an irreversible denatured structure [74] (Figure 4b). Similar to triosephosphate isomerase, heat treatment at temperatures higher than 60 °C resulted in structural disruption an a decrease in the IgE-binding activity of filamin C [74] (Figure 4b). Arginine kinase is also a thermal-sensitive allergen which aggregates in a temperature higher than 44 °C, while increased IgE-binding activity of AK can be observed at 44–70 °C. Continuous increasing heat led to IgE-binding activity gradually decreasing, and ultimately being eliminated when temperatures were 80 °C or higher [55]. Arginine kinase contains free sulfhydryl in its molecules, and it can be speculated that the free sulfhydryl participates in aggregation by disulfide. Arginine kinase is enriched upon aggregation, thus increasing IgE-binding activity (Figure 4c).

It is worth noting that all the studies came to an understanding that crustacean tropomyosin retains great allergenicity even after heat treatment. For instance, in a study by Chen et al., tropomyosin from eight randomly selected fish species showed the most stable existence and immunoreactivity among all the muscle proteins [91]. Tropomyosin from *Crassostrea gigas* showed a significantly higher IgE reactivity after roasting and boiling processing, with roasted protein having slightly more IgE reactivity than the boiled one [92]. The authors then explored the mechanism from the aspect of structure and found that the two-stranded α-helical coiled-coil structure of tropomyosin was destroyed and gradually converted to a single-stranded α-helix, which led to an altering of the hydrophobic face that might contain IgE-binding epitopes (Figure 4d). Moreover, the IgE binding of tropomyosin in extracts of shrimp processed in different ways, including boiling, steaming, baking, frying, and microwaving, was also higher than that of a raw sample [93]. Another thermostable allergen, sarcoplasmic calcium-binding protein, showed virtually invisible immunoblot band intensity after heat treatment by the same methods, despite the detectable band in SDS-PAGE [94]. It seems that there is a variety of IgE-binding activity with respect to sarcoplasmic calcium-binding protein subject to different thermal treatment conditions. Sarcoplasmic calcium-binding protein retained immunological binding capacity in a water bath at 30–100 °C despite structural changing and polymer formation [72]. Moreover, myosin light chain 2 has also shown high resistance to boiling [64], while further condition optimization and understanding of in-depth mechanisms are yet to be fulfilled.

Microwave heating is a novel thermal treatment involving the interaction of microwaves and the medium by volumetric dissipation of electromagnetic energy in the form of heat. Microwaves travel through the lossy medium, producing an increase in medium temperature [95]. Thus, microwaves are able to affect the kinetics of conformational changes to allergens and to accelerate their denaturation. Despite some attempts to use microwaves to reduce the allergenicity of food [93], techniques that combine microwave heating with other processing methods are necessary in order to eliminate the antigenicity of proteins.

#### 3.1.2. Non-Thermal Treatments

**Figure 5:**
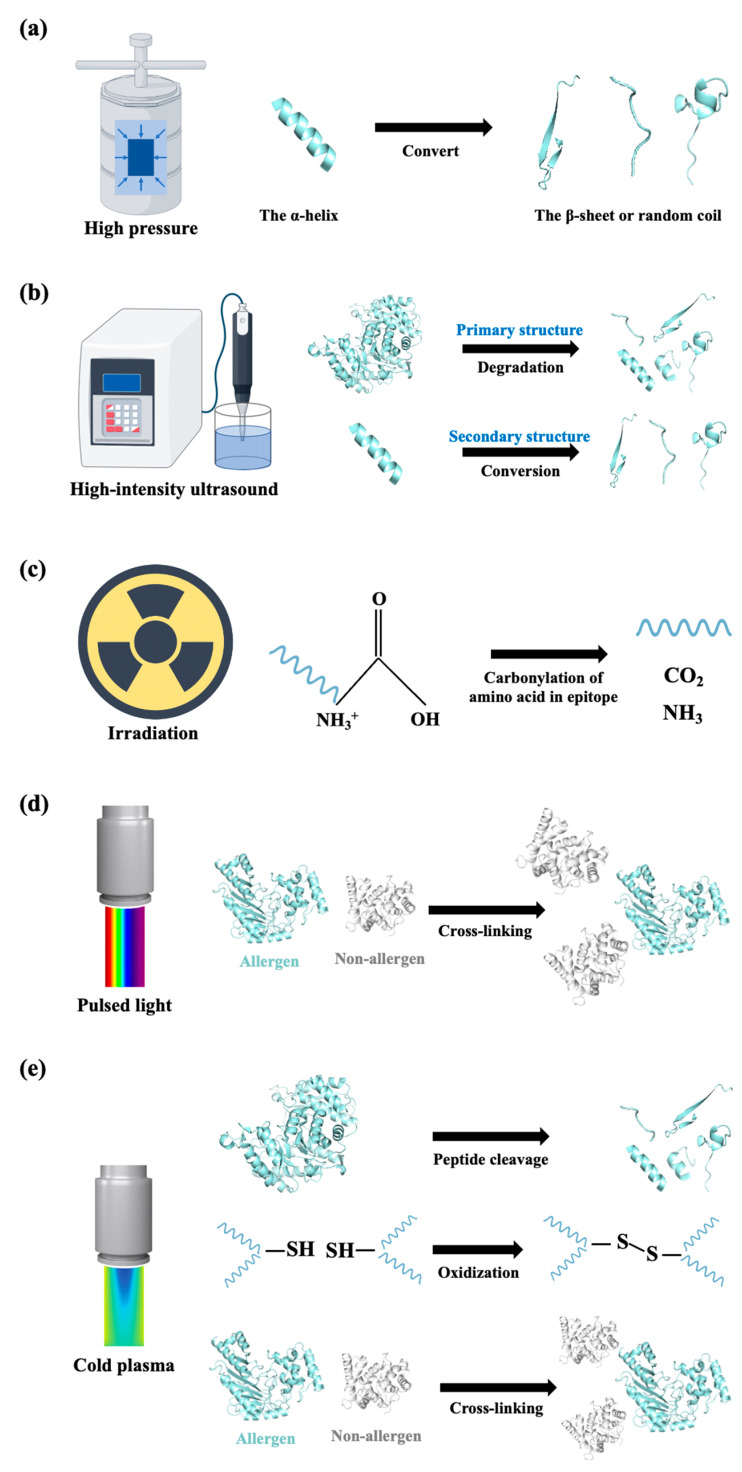
Schematic representation of innovative non-thermal processing methods in terms of structural changes to seafood allergens. (**a**) Secondary structure conversion during high-pressure treatment; (**b**) primary structure degradation and secondary structure conversion of allergen induced by high-intensity ultrasound treatment; (**c**) carbonylation of amino acids in epitopes during irradiation; (**d**) allergenic proteins cross-linking with heat-sensitive proteins during pulsed ultraviolet light treatment; (**e**) cleavage of allergen peptide bonds, oxidization of amino acid side residuals, and formation of protein–protein cross-linkages during cold plasma treatment. All structural information related to allergens was sourced from the RCSB Protein Data Bank (PDB; https://www.rcsb.org/). The instruments were sourced from professional scientific illustration platform Figdraw (https://www.figdraw.com/).

Thermal treatments were found to be inefficient in terms of altering the IgE-binding ability of thermostable allergens, and were even shown to markedly increase the IgE reactivity of tropomyosin. To minimize the negative effects of thermal processing techniques, innovative non-thermal techniques are sought to reduce or eliminate the allergenicity of seafood. High-pressure processing involves pressure of 100 MPa to 1000 MPa for a shorter duration of time, which gives rise to structural distortion of a protein by changing the non-covalent bonds [96] (Figure 5a). In a study by Jin et al. [97], tropomyosin from *Todarodes pacificus* was treated with high hydrostatic pressure of 200, 400, and 600 MPa at 20 °C. The structure of tropomyosin denatured via a conversion of α-helix to β-sheet and random coils and via a decreasing of the free sulfhydryl group. This structural distortion resulted in a decreasing of digestibility of tropomyosin and, thus, reduced its allergenicity [97]. Electrophoresis analysis revealed that high-pressure processing decreased the SDS-PAGE band intensity of fish allergens, including enolase, creatine kinase, aldolase, and triosephosphate isomerase, when the pressure reached 430 MPa, while the existence of IgE-binding fragments has not been determined [98]. It is worth noting that the intensity of parvalbumin was found to increase at this pressure. Recently, the application of high hydrostatic pressure treatment of 200–400 MPa was found to successfully change the structure of parvalbumin, which suggests the potential application of high hydrostatic pressure treatment in terms of reducing the allergenicity of parvalbumin [99].

High-intensity ultrasound uses high-energy mechanical waves (20–100 kHz), which can cause the breaking of chemical bonds and the occurrence of the redox reaction of free radicals, leading to covalent bonds breaking and molecular degradation [100] (Figure 5b). By treating with high-intensity ultrasound (20 kHz, 100–800 w) for 15 min, the allergenicity of *Exopalaemon modestus* tropomyosin was significantly reduced [101]. It was demonstrated that the ultrasound influenced the primary, secondary, and tertiary structure of tropomyosin: at the amino acid level, ultrasound treatment oxidized the cystine, methionine, and lysine in tropomyosin and degraded it to generate protein fragments; in the secondary structure, a conversion of α-helix to β-sheet, β-turn, and random coil occurred, resulting in a loosened tertiary structure [101] (Figure 5b). Li et al. reported that ultrasound (30 kHz, 800 W, 1.5 h) at 0 °C and 50 °C slightly reduced the allergenicity of raw shrimp; however, allergenicity reduction by ultrasound treatment with respect to boiled shrimp samples was more effective than ultrasound treatment of raw samples [102]. It is interesting to note that the most reduced allergenicity for tropomyosin was found in the samples treated by ultrasound (30 kHz, 800 W, 1.5 h) at 0 °C [102]. It seemed that there were several allergens in the muscle extract that participate in its allergenicity, indicating that it is more comprehensive to optimize processing conditions for extracts than for purified allergens.

Irradiation has been regarded as a cold pasteurization practice for preserving food with minimal alteration to the nutritive and sensorial characteristics of foods [103]. During irradiation, the energy from γ-rays, X-rays, or electron beams is directly absorbed, leading to changes in the spatial structure and conformation of the allergenic proteins, thereby reducing their allergenicity [104]. It was found that under the conditions of irradiation with varying doses (1–13 kGy) by electron beam irradiation at 10 MeV, the IgE-binding activity of turbot parvalbumin decreased markedly upon increasing the irradiation dose [105]. The author speculated that the decreasing IgE-binding activity resulted from the carbonylation of amino acids in epitopes, according to the observation that protein carbonyl content has a positive relation with IgE-binding capacity reduction (Figure 5c). Electron beam irradiation treatment is also efficient at eliminating the allergenicity of shrimp tropomyosin. Guan et al. demonstrated that irradiation treatment broke the hydrogen bonds in tropomyosin, which brought a more extended structure of protein accompanied by epitope disruption, thus reducing the binding activity of tropomyosin with specific antibodies [106]. Moreover, paramyosin and myosin heavy chain contents of the myofibrillar protein from *Tegillarca granosa* were partially degraded by irradiation treatment [107], indicating the capacity of irradiation treatment to eliminate the allergenicity of thermostable allergens and allergens from the actin-binding family.

Pulsed light and cold plasma are two novel processing techniques that are able to decrease seafood allergenicity by altering the conformation of allergens (Figure 5d,e). Pulsed light delivers wavelengths from 200 nm to 1000 nm. Pulsed light is formed by short and intensive pulses of white light with a broad spectrum and is made up of 54% ultraviolet light, 20% infrared, and 26% visible light radiation [108]. Such efficient pulsed ultraviolet light can ionize proteins by absorbing photons, such as the absorption and recombination of aromatic amino acids. Pulsed ultraviolet light induces allergenic protein cross-linking with some heat-sensitive proteins and further leads to allergenicity reduction [32] (Figure 5d). Cold plasma is in a gaseous state and is generated at 30–60 °C under atmospheric or vacuum pressure. It changes the protein conformation, presumably by the cleavage of peptide bonds, the oxidization of amino acid side residuals, and the formation of protein–protein cross-linkages, which further induce the effectively lower IgE-binding activity of allergens [109] (Figure 5e). Cold plasma has been applied to the elimination of shrimp tropomyosin. An increase in surface hydrophobicity and a decrease in free sulfhydryl were observed in tropomyosin with a longer treatment time [110]. Current studies show the promising performance of pulsed light and cold plasma in reducing allergenicity, and follow-up studies are now focusing on better setup parameters and combining these methods with other technologies.

### 3.2. Chemical Processing Techniques

In recent years, enzymatic-catalyzed cross-linking and glycation modification (Maillard reaction and enzymatic-catalyzed glycation) are preferred as effective, selective, energy-saving, and eco-friendly techniques in terms of processing hypoallergenic food.

#### 3.2.1. Enzymatic-Catalyzed Cross-Linking

Cross-linking of proteins has been exploited in the food industry for cereal, dairy, meat, and fish processing as a means of stabilizing food structure/texture and improving food functionality [111]. In addition to its textural-modifying effects, the cross-linking of food proteins can effectively conceal IgE-binding epitopes, consequently reducing the allergenic potential of food products [112,113]. Fei et al. reported that polymerization with tyrosinase of crab arginine kinase affected the action of the gastrointestinal enzymes, and it presented lower antigenic properties than those of the untreated protein [114]. The IgE-binding activity of crab tropomyosin was proved to be mitigated by 63.5% and 34.5% through cross-linking by tyrosinase and horseradish peroxidase, respectively [115]. It is worth nothing that polymerization of tropomyosin with horseradish peroxidase facilitated the action of the gastrointestinal enzymes, and its cross-linking product not only has the potential to reduce allergenicity, but also has the capacity to induce oral tolerance in mice [115]. Allergenicity elimination of tropomyosin can also be found in tropomyosin cross-linking by tyrosinase and laccase in a dose-dependent manner, due to the changes in the structural integrity of the allergenic protein [113,116]. Tian et al. reported that polymerization of fish parvalbumin with tyrosinase facilitated the action of the gastrointestinal enzymes, and it presented lower antigenic properties than those of the untreated protein [117]. Tyrosinase polymerization has also been performed by Hu et al. in order to cross-link crab sarcoplasmic calcium-binding protein for allergenicity elimination [72].

**Figure 6:**
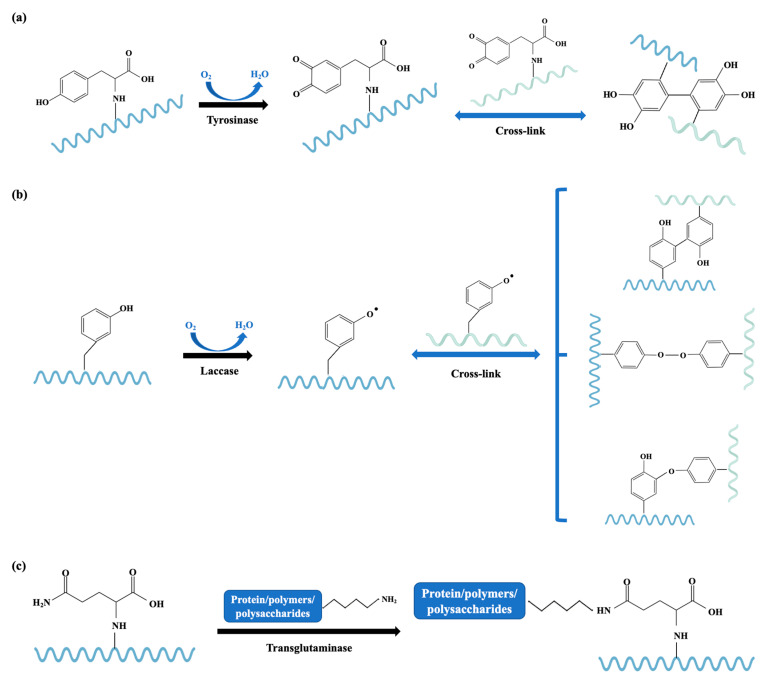
Schematic diagram of the catalytic mechanism of tyrosinase (**a**), laccase (**b**), and transglutaminase (**c**). The different polypeptide chains are shown in wavy lines with different colors.

It can be found from the above examples that the ultimate reactions of enzymatic cross-linking are achieved depending on the enzyme used, the availability of the target reactive groups, and the applied process conditions. Tyrosinase is an oxidase that catalyzes the oxidation of tyrosine and results in oxidative cross-linking of tyrosine side chains [118]. Tyrosine in an allergen can be catalyzed by tyrosinase to form new bonds between tyrosine residues and reactive benzoquinone intermediates in proteins, which may introduce both intra- and intermolecular covalent cross-links, thus changing and masking the allergic epitopes (Figure 6a). Laccase is another commonly used oxidase that catalyzes the oxidation of a wide range of phenolics and related compounds, resulting in the formation of unstable aromatic radicals that react with proteins, leading to cross-linked products [113] (Figure 6b). With the help of the hydroxyl functional group provided by caffeic acid, the cross-linking efficacy of oxidative enzymes can be augmented [113,114]. The cross-linking of tropomyosin by tyrosinase was not as efficient at allergenicity elimination as peroxidase because of the different structure of the cross-linked tropomyosin: cross-linking via peroxidase resulted in a product with higher hydrophilicity than that of tyrosinase, which makes it easier for pepsin to find cleavage sites [115]. Transglutaminase is responsible for acyl transfer, deamidation, and crosslinking of intra- or inter-chain glutamine peptide moiety, which is the acyl donor and lysine peptide moiety, and also the acyl acceptor [119] (Figure 6c). The modifications of the allergenic proteins using enzyme-catalyzed protein cross-linking offer mild and safe approaches to mitigate the allergenicity of seafood, while more investigations with respect to allergen structure and epitopes are necessary to make full use of this technique.

#### 3.2.2. Glycation Modification

**Figure 7:**
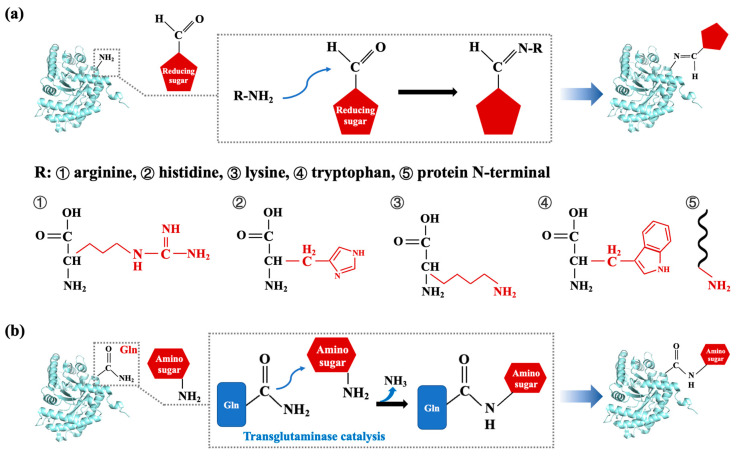
The reaction principle of glycation modification. (**a**) Overview of the key steps of the Maillard reaction, with the side chains of potential amino acids in the Maillard reaction marked in red; (**b**) overview of the key steps of enzymatic-catalyzed glycation, illustrated by the reaction of amino sugar with a glutaminyl residue that is catalyzed by transglutaminase.

Glycation modification is considered a method of changing food allergenicity with mild reaction conditions, low cost, and improved functional properties of proteins. Non-enzymatic glycation, also referred to as the Maillard reaction, involves a non-enzymatic browning reaction between the amino acid residue of proteins and reducing sugars [120]. By introducing sugar chains to the free amino groups on proteins, usually lysine, arginine, and N-terminal amino acids, the Maillard reaction can modify, destroy, or mask sequential epitopes, result in changes in conformational epitopes, or even cause damage to the structure of an allergen (Figure 7a). Previous studies have demonstrated that the impact of the Maillard reaction on food allergenicity is varied and relies on the structural properties of allergens, the types and amounts of reducing sugars, and treatment conditions (e.g., temperature, pH, and moisture).

Most of the work related to allergenicity elimination through the Maillard reaction are targeted at shellfish tropomyosin. According to Han et al., crab tropomyosin glycated by galactose, glucose, and arabinose modified the arginine and lysine in the epitope as well as varied the protein structure, resulting in decreasing allergenicity [121]. A similar result was supported by the work of Zhang et al., where shrimp tropomyosin reacted with either glucose, maltose, or maltotriose; after being glycated, lysine in shrimp tropomyosin, especially in the epitopes, was modified, and the products showed lower IgE-binding capacity and induced weaker allergy symptoms in vivo [122]. The utilization of distinct reducing sugars in the Maillard reaction induces differential modifications of amino acid residues, with the specific chemical alterations being contingent upon the structural characteristics of both the saccharide and amino acid involved. Lv et al. incubated shrimp tropomyosin with ribose, which led to modifications in the phenylalanin, isoleucine, and methionine residues of epitopes; the IgE-binding capacity of tropomyosin was decreased for the masked or destroyed epitopes [123]. In a recent work by Zhao et al., the Maillard reaction between shrimp tropomyosin and different kinds of reducing sugars was found to sharply decrease the IgG/IgE-binding activity of allergens with different mechanisms [124]. Glucose, ribose, and lactose modified the lysine and arginine in the epitopes, while the trehalose and chitosan molecules formed a non-covalent interaction with tropomyosin.

The Maillard reaction was also found to be efficient at reducing the allergenicity of tropomyosin in mollusks. The IgE-binding activity of tropomyosin in *Octopus fangsiao* can be reduced by approximately 60% compared with untreated protein after a 24 h reaction with glucose. It was speculated that the glucose added to lysine and arginine residues of tropomyosin altered the cut sites of trypsin and chymotrypsin [125]. Under different reaction conditions, xylose decreased theimmunobinding activity of tropomyosin from both *Chlamys nobilis* and *Haliotis discus hannai* in different ways. The Maillard reaction between scallop tropomyosin and xylose in a dry state led to the modification of lysine and arginine, and epitopes were destroyed and masked by saccharide residues on the tropomyosin surface [126]. In a wet-state Maillard reaction, the xylose tended to modify methionine and asparagine in linear epitopes of abalone tropomyosin, which resulted in IgE-binding capacity being decreased by approximately 70% [127]. There are some other allergens in seafood where the Maillard reaction resulted in allergenicity changes. Arginine kinase from *S. paramamosain* reacting with arabinose was found to present lower allergenicity, both in vitro and in vivo, via the modification of arginine and lysine in epitopes, accompanied by a variation in spatial structure [72,128].

Recently, significant attention has been paid to enzymatic-catalyzed glycation that can site-specifically link the sugar at lower temperatures due to its strict substrate specificity. Transglutaminase can induce an acyl-transfer reaction between the γ-carboxyamide of glutaminyl residues and primary amines; thus, amino sugars can be regarded as a substrate for the cross-linking of protein and sugar [129] (Figure 7b). It was demonstrated that glutamine, lysine, and arginine containing free amino groups were involved in enzymatic cross-linking reactions, which affected the structure of tropomyosin and altered its allergenicity [127,130,131]. These investigations revealed the glutamine and lysine in epitopes were modified by glucosamine, and further glycation might also lead to structural variation in the allergen, thus reducing its IgE-binding activity. As compared with other methods, such as heating or the Maillard reaction, enzymatic-catalyzed glycation can induce site-specific modification under mild conditions. Transglutaminase-catalyzed glycation between allergen and glucosamine appears to be useful for reducing the allergic hazards of seafood in the food industry.

### 3.3. Biological Processing Techniques

Several studies highlighted the use of biological food processing technologies as a potential allergen mitigation strategy. In terms of reducing food allergenicity, biological processing is mainly divided into enzymatic hydrolysis and fermentation treatment [132].

#### 3.3.1. Enzymatic Hydrolysis

Enzymatic hydrolysis via proteolytic enzymes is a traditional and effective way to modify the allergenicity of allergens [112,113]. Enzymatic hydrolysis at a low degree can partially cleave the protein and lead to the disrupture of conformational and linear epitopes, and a greater degree of hydrolysis results in the degradation of amino acid sequences, which cuts the allergens into small fragments [112]. The proteinases utilized for the preparation of protein hydrolysates can be found in different sources, such as gastric enzymes (e.g., pepsin, trypsin, and chymotrypsin), plant-derived enzymes (e.g., papain and bromelain), and microbial-derived enzymes (e.g., alcalase and substilisin) [133].

Untersmayr et al. reported that under pepsin digestion at a pH < 2.5, all proteins of codfish extract including parvalbumin were degraded to small fragments within 1 min and lost their IgE-binding capability [134]. With an increased gastric enzyme/protein ratio, crustacean tropomyosin was also degraded into small fragments and underwent notable decreasing in IgE-binding sites [135,136]. The findings by Keshavarz et al. demonstrated the similar digestion characteristics of fish tropomyosin with respect to crustacean tropomyosin, with the allergenicity of tropomyosin gradually decreasing as a function of pepsin digestion time. However, allergens digested in the gastrointestinal tract are not directly equivalent to an elimination of allergenicity [137]. However, a number of seafood allergens, such as arginine kinase, triosephosphate isomerase, filamin C, myosin light chain, and sarcoplasmic calcium-binding protein, can be digested in the gastrointestinal tract while the digestion products retain their IgE-binding activity [49]. García-Moreno et al. evaluated the impact of different enzymatic treatments (subtilisin, trypsin, and a combination of both) on the allergenicity of parvalbumin from *Micromesistius poutassou* and found that subtilisin hydrolysates present the lowest allergenicity [138]. It has been reported that enzymes used singly or in combination could be effective to develop hypoallergenic milk and oyster products [133,139], indicating that modifications of the allergenic proteins using proteolytic hydrolysis may offer new approaches to mitigate the allergenicity of seafood allergens.

#### 3.3.2. Fermentation Treatment

Fermentation takes place when microorganisms act on food substrates. Fermentation plays a crucial role in improving food quality and microbiological stability in the food industry. During fermentation, microbial enzymatic activity modifies or disrupts allergenic epitopes in food substrates, leading to reduced allergen immunoreactivity. Current evidence suggests that fermentation mitigates food allergenicity predominantly by three routes: (1) structures and epitopes are destroyed due to proteolysis and acid-induced denaturation; (2) epitopes are masked by glycosylation and Maillard reactions that occurred during fermentation; (3) some allergens are released into the water [140].

Previous research has shown that among various industrial processing methods, fermentation is one of the most effective methods for reducing the IgE-binding capacity of fish products [141]. Zou et al. observed the changes in allergenicity of *Atlantic cod* treated by fermentation with *Lactobacillus helveticus* in a salt concentration of 2% at 30 °C for 60 h. The study reported that the immunoreactivity of parvalbumin decreased after fermentation due to protein hydrolysis, leading to the destroying of epitopes [142]. Similar results have also been documented by Zhu et al., where the IgG/IgE-binding activity of *Mylopharyngodon piceus* muscle can be decreased by fermentation processing [143]. For shellfish, fermentation processing has also been demonstrated to effectively mitigate allergenicity. The manufacturing process of fermented shrimp paste induces gradual protein degradation, particularly affecting tropomyosin, which consequently diminishes the IgE-binding activity of the product [144]. It can be concluded that fermentation processing can be used to reduce seafood allergenicity. Moreover, fermentation, compared to other processing techniques, is able to mitigate food allergenicity without being limited for safety reasons (e.g., irradiation) and by high costs (e.g., high pressure). Nevertheless, given the complexity of fermentation parameters, clarifying the precise mechanisms underlying allergenicity reduction has proven particularly challenging.

### 3.4. Combination Processing

Combined treatments probably show a better performance in reducing allergenicity. Studies have applied a combination of physical and chemical processing methods to lower seafood allergenicity, normally including combined high temperature with high pressure, combined high temperature with enzymatic-catalyzed cross-linking, and combined non-thermal treatment with glycation.

Tropomyosin maintains its activity even after being boiled in water. Long et al. treated shrimp tropomyosin with high hydrostatic pressure (500 MPa and 55 °C for 10 min); the product exhibited significant loss of allergenicity. And by combining high hydrostatic pressure with thermal treatment (55–75 °C), the allergenicity of tropomyosin was thoroughly eliminated [145]. By combining the Maillard reaction (with galactose) with high temperature and pressure (0.08 MPa, 115 °C), the IgE-binding activity of shrimp meat can also be significantly decreased [146]. Moreover, the lysine and arginine in the epitopes of sarcoplasmic calcium-binding protein were modified by galactose during the Maillard reaction, and the high temperature and pressure can further alter thermostable allergens, for instance, by breaking the disulfide bonds [146]. Fei et al. found that the singly processed crab arginine kinase was relatively stable without significant alteration of IgE-binding activity after heating (50 °C, 30 min) and cross-linking (tyrosinase, 37 °C, 8 h) [114]. However, the combined treatment of heating (50 °C, 30 min) and cross-linking (tyrosinase, 37 °C, 8 h) significantly reduced the intensity of the allergenicity of arginine kinase, as determined via inhibition ELISA analysis and in vivo studies, respectively [114].

A microwave cooking-assisted Maillard reaction (with glucose) was found to be a better way to reduce the immunogenicity and immunoreactivity of parvalbumin than the traditional heating method. Microwave heating can speed up the chemical synthesis reaction and the shorten reaction time from several days to several minutes. The lysine, threonine, and aspartic acid in epitopes of glycated parvalbumin were modified by glucose; meanwhile, Ca^2+^-binding sites were destroyed in the microwave-assisted Maillard reaction, which affected parvalbumin allergenicity [147]. In addition, combined Maillard reaction and non-thermal processing techniques with respect to allergen immunogenicity are also reported. Zou et al. reported that cold plasma combined with the Maillard reaction (with ribose) efficiently alters the IgE-binding capacity of shrimp tropomyosin [148]. The reduction was found to be associated with the combined effects: modification induced by cold plasma destroyed the core helical structure of tropomyosin and occupied the potential glycation sites, leading to sequential glycation on conserved areas of tropomyosin [148]. Additionally, the destroyed structure exposed an increasing number of cleavage sites of trypsin, which may result in lower digestibility and reduced IgE-binding capacity of digestion products.

Compared to the single treatments, the combination of different processing techniques leads to a more significant effect in terms of decreasing seafood allergenicity, because reductions in allergenicity benefit from the different mechanisms of the various processing methods. However, studies investigating combined treatment methods for seafood processing are still insufficient. Information on the epitopes and structural properties of allergens is the basis for choosing, and optimizing of, the appropriate combined processing methods. Research involving combined processing techniques should take the property of allergens into consideration when optimizing processing conditions via orthogonal designed experiments.

## 4. Challenges and Future Directions

Allergic reactions triggered by seafood consumption represent a worldwide public health issue. Food processing has been widely recognized as a practical and efficient method for reducing allergenicity by inducing structural alterations or epitope modifications in allergens. However, despite its demonstrated efficacy, significant challenges remain in terms of fully characterizing processing-induced structural modifications and elucidating the fundamental mechanisms involved.

Firstly, the molecular foundation of allergic responses lies in the specific interaction between IgE antibodies and their cognate epitopes [149]. Detailed information on epitopes provides crucial insights for elucidating the molecular mechanisms underlying allergenicity reduction through food processing. Chemical-based and biological-based processing methods—enzymatic glycosylation, the Maillard reaction, and enzymatic hydrolysis—all function by selectively modifying or cleaving specific amino acid residues within allergen molecules, thereby altering the structure, epitopes, and allergenicity of allergens. The amino acid profile of epitopes can serve as a selection criterion for optimal enzymes/reducing sugars in these processing techniques, consequently enhancing allergen mitigation efficacy. Processing technologies targeting epitope modification might become an important research direction, while the number of characterized epitopes remains limited compared to the actual epitope repertoire, with conformational epitopes being particularly underrepresented. Furthermore, the structural reorganization and conformational shifts occurring during food processing of some seafood allergens can effectively diminish their allergenic potential. Under these conditions, comprehensive structural characterization of allergens is required to establish robust structure–allergenicity relationships and advance mechanistic understanding. Given the structural homology among allergens within the same protein family [150], processing strategies targeting their shared structural motifs could potentially mitigate the allergenicity of multiple allergens concurrently. These findings highlight the critical need for further structural investigations of seafood allergens to advance our understanding. Moreover, in most previous studies, purified allergens have been subjected to food processing that exclude the potential impact of the food matrix; however, growing evidence suggests that food processing can induce physicochemical interactions between food matrix components and allergens [151]. These interactions may have dual effects: processing may promote the aggregation of allergens with matrix components, thereby hindering the degradation of allergenic epitopes; alternatively, it can facilitate chemical reactions between allergens and matrix constituents, potentially leading to the formation of novel allergenic compounds [152]. Consequently, the complexity of food matrices should be considered in practical production processes. In addition, current investigations into processed seafood allergenicity primarily rely on preclinical data obtained through serological assays, cellular models, and murine systems, and critical clinical evidence derived from seafood-allergic patients remains insufficient.

## 5. Conclusions

Many attempts have been made to identify seafood allergens and clarify the epitopes and structure–allergenicity relationship. Accumulating knowledge regarding allergen structures and epitope profiles has provided critical insights into the molecular basis of food processing-mediated allergenicity mitigation. From a structural–immunological perspective, food processing, particularly when mediated by physical methods, induces conformational modifications in allergenic proteins that ultimately diminish their immunoreactive potential. From an epitope perspective, chemical processing techniques can induce covalent or non-covalent interactions between allergens and various modifiers (enzymes, saccharides, or other allergens), effectively masking critical epitopes through molecular complexation. Biological processing induces substantial structural modifications in allergens, including protein unfolding, polypeptide chain uncoiling, and enzymatic degradation, which collectively alter conformational epitopes and significantly attenuate allergenic potential. Future investigations should prioritize elucidating structure–allergenicity relationships to clarify the fundamental mechanisms underlying allergenicity reduction. This mechanistic understanding will subsequently facilitate the development of innovative processing technologies for effective seafood allergen mitigation or elimination.

## Figures and Tables

**Table 1:** Allergens identified in fish (with data from the WHO/IUIS http://www.allergen.org).

Protein	Fish Species	Allergen Names in IUIS	MW (kDa)
β-Parvalbumin	*Gadus callarias*	Gad c 1	12
	*Gadus morhua*	Gad m 1	12
	*Crocodylus porosus*	Cro p 1	11.6
	*Clupea harengus*	Clu h 1	12
	*Ctenopharyngodon idella*	Cten i 1	9
	*Cyprinus carpio*	Cyp c 1	12
	*Lates calcarifer*	Lat c 1	11.5
	*Lateolabrax maculatus*	Late m 1	12 and 13
	*Lepidorhombus whiffiagonis*	Lep w 1	11.5
	*Oncorhynchus mykiss*	Onc m 1	12
	*Pangasianodon hypophthalmus*	Pan h 1	11
	*Rastrelliger kanagurta*	Ras k 1	11.3
	*Salmo salar*	Sal s 1	12
	*Sardinops sagax*	Sar sa 1	12
	*Scomber scombrus*	Sco s 1	12
	*Sebastes marinus*	Seb m 1	11
	*Solea solea*	Sole s 1	11~14
	*Thunnus albacares*	Thu a 1	11
	*Trichiurus lepturus*	Ttic l 1	11
	*Xiphias gladius*	Xip g 1	11.5
α-Parvalbumin	*Crocodylus porosus*	Cro p 2	13
β-Enolase	*Gadus morhua*	Gad m 2	47.3
	*Cyprinus carpio*	Cyp c 2	47
	*Pangasianodon hypophthalmus*	Pan h 2	50
	*Salmo salar*	Sal s 2	47.3
	*Thunnus albacares*	Thu a 2	50
Aldolase A	*Gadus morhua*	Gad m 3	40
	*Pangasianodon hypophthalmus*	Pan h 3	40
	*Salmo salar*	Sal s 3	40
	*Thunnus albacares*	Thu a 3	40
Tropomyosin	*Salmo salar*	Sal s 4	37
	*Oreochromis mossambicus*	Ore m 4	33
	*Pangasianodon hypophthalmus*	Pan h 4	35
β-Prime-component of vitellogenin	*Oncorhynchus keta*	Onc k 5	18
α-Collagen	*Lates calcarifer*	Lat c 6	130~1140
	*Salmo salar*	Sal s 6	130~140
Creatine kinase	*Pangasianodon hypophthalmus*	Pan h 7	43
	*Salmo salar*	Sal s 7	43
Triosephosphate isomerase	*Pangasianodon hypophthalmus*	Pan h 8	25
	*Salmo salar*	Sal s 8	25
Pyruvate kinase PKM-like	*Pangasianodon hypophthalmus*	Pan h 9	65
L-lactate dehydrogenase	*Pangasianodon hypophthalmus*	Pan h 10	34
Glucose 6-phosphate isomerase	*Pangasianodon hypophthalmus*	Pan h 11	60
Glyceraldehyde-3-phosphate dehydrogenase	*Pangasianodon hypophthalmus*	Pan h 13	36

Note: The allergens were arranged in accordance with their allergen names in the IUIS list.

**Table 2:** Allergens identified in fish and shellfish and their remarkable characteristics (with data from the WHO/IUIS http://www.allergen.org).

Protein	MW (kDa)	Species	Biological Function	Remark
β-Parvalbumin	11~14	Fish	Calcium-binding protein	• Thermostable • The Ca^2+^ is important for physiological conformation and IgE-binding activity • Most of the IgE-binding epitopes are located on the loop regions between α-helices • Key epitope: AA 95~108
α-Parvalbumin	~13	Fish	Calcium-binding protein	• Thermostable • Allergenicity of α-parvalbumin is generally considered to be lower than β-parvalbumin
β-Enolase	47~50	Fish	Glycolytic enzyme	• Thermal sensitive
Aldolase A	40	Fish	Glycolytic enzyme	• Thermal sensitive
Tropomyosin	33~40	Fish Crustacean Mollusk	Actin-binding protein	• Resistance to heating or acidic conditions • Heat/digested stable regions: AA 19–29, 39–48, 77–82, 99–109, 110–123, 134–139, 153–162, 170–188, 211–221, 250–254, 267–274
Collagen	130~140	Fish	Structural protein	• Resistance to heating; becomes water soluble and acquires higher allergenicity after heat treatment
Pyruvate kinase PKM-like	65	Fish Crustacean	A regulation enzyme involved in glycolysis	• Resistance to heating
Triosephosphate isomerase	25~28	Fish Crustacean Mollusk	A key enzyme involved in glycolysis	• Thermal sensitive • Resistance to acidic and alkaline conditions
Creatine kinase	43	Fish	Phosphoryl transfer enzymes	• Thermal sensitive
L-lactate dehydrogenase	34	Fish	An enzyme involved in glycolysis	-
Glucose 6-phosphate isomerase	60	Fish	A housekeeping enzyme of glycolysis and gluconeogenesis	-
Glyceraldehyde-3-phosphate dehydrogenase	36	Fish	-	-
β-Prime-component of vitellogenin	18	Fish	-	-
Arginine kinase	38~45	Crustacean Mollusk	Phosphoryl transfer enzymes	• Thermal sensitive • Intramolecular disulfide bond Cys_201_-Cys_271_ is integral for maintaining conformation • Hotspot linear/conformational epitope region: α-helical N-terminal domain • Dominating linear epitope regions: AA 125–187, AA 232–250, and AA 302–32 • Heat/digested stable regions: AA 34–47, 61–68, 91–97, 101–117, 159–166, 179–195, 230–243, 246–256, 294–304, 310–323, 340–349
Paramyosin	99	Mollusk	Actin-binding protein	• Resistance to acidic and alkaline conditions • Relatively stable during heating • Capable to bind Ca^2+^
Myosin light chain 2	18~23	Crustacean	Actin-binding protein	-
Myosin light chain 1	17.5~18	Crustacean	Actin-binding protein	• Resistance to heating and extreme pH • Heat/digested stable regions: AA 39–48, 66–73, 77–87, 109–126, 136–145
Sarcoplasmic calcium-binding protein	20~25	Fish Crustacean Mollusk	Calcium-binding protein	• Resistance to heating and extreme pH • The Ca^2+^ is important for physiological conformation and IgE-binding activity • Several epitopes were found to be located in the Ca^2+^-binding sites • Heat/digested stable regions: AA 39–51, 77–82, 111–120, 139–149
Filamin C	~90	Crustacean	Actin-binding protein	• Cross-reactive with triosephosphate isomerase • Sensitive to heat and extreme pH • Digested stable peptides might exist
Troponin C	16.8~21	Crustacean	Actin-binding protein	• Capable of binding to Ca^2+^
Troponin I	~30	Crustacean	Actin-binding protein	-
Hemocyanin	76	Crustacean	-	• Cross-reactive with insect allergen
Ovary development-related protein	28.2	Crustacean	-	-
Cytoplasmic fatty acid-binding protein	15~20	Crustacean	Lipid binding protein superfamily	• Cross-reactive with cockroach allergen Der p 13
Glycogen phosphorylase-like protein	95	Crustacean	-	-

Note: The allergens were arranged in accordance with their mentioned order in the text.

**Table 3:** Allergens identified in shellfish (data from the WHO/IUIS http://www.allergen.org).

Protein	Shellfish Species	Allergen Names in IUIS	MW (kDa)
Tropomyosin	*Charybdis feriatus*	Cha f 1	34
	*Crangon crangon*	Cra c 1	~38
	*Crassostrea angulata*	Cra a 1	38
	*Crassostrea gigas*	Cra g 1	38
	*Exopalaemon modestus*	Exo m 1	38
	*Haliotis laevigata x Haliotis rubra*	Hal l 1	33.4
	*Helix aspersa*	Hel as 1	36
	*Homarus americanus*	Hom a 1	34
	*Litopenaeus vannamei*	Lit v 1	36
	*Macrobrachium rosenbergii*	Mac r 1	37
	*Melicertus latisulcatus*	Mel l 1	38
	*Metapenaeus ensis*	Met e 1	34
	*Pandalus borealis*	Pan b 1	37
	*Panulirus stimpsoni*	Pan s 1	34
	*Penaeus aztecus*	Pen a 1	36
	*Penaeus indicus*	Pen i 1	34
	*Penaeus monodon*	Pen m 1	38
	*Portunus pelagicus*	Por p 1	36
	*Procambarus clarkii*	Pro c 1	36
	*Saccostrea glomerata*	Sac g 1	38
	*Scylla paramamosain*	Scy p 1	38
	*Todarodes pacificus*	Tod p 1	15
Arginine kinase	*Callinectes bellicosus*	Cal b 2	40
	*Crassostrea angulata*	Cra a 2	38~41
	*Crangon crangon*	Cra c 2	~45
	*Litopenaeus vannamei*	Lit v 2	40
	*Macrobrachium rosenbergii*	Mac r 2	40
	*Penaeus monodon*	Pen m 2	40
	*Procambarus clarkii*	Pro c 2	40
	*Scylla paramamosain*	Scy p 2	40
Ovary development-related protein	*Eriocheir sinensis*	Eri s 2	28.2
Paramyosin	*Rapana venosa*	Rap v 2	99
Myosin light chain 2	*Homarus americanus*	Hom a 3	~23
	*Litopenaeus vannamei*	Lit v 3	20
	*Penaeus monodon*	Pen m 3	20
	*Scylla paramamosain*	Scy p 3	18
Myosin light chain 1	*Artemia franciscana*	Art fr 5	~17.5
	*Crangon crangon*	Cra c 5	~17.5
	*Procambarus clarkii*	Pro c 5	18
Sarcoplasmic calcium-binding protein	*Crangon crangon*	Cra c 4	~25
	*Crassostrea angulata*	Cra a 4	20
	*Litopenaeus vannamei*	Lit v 4	20
	*Penaeus monodon*	Pen m 4	20
	*Pontastacus leptodactylus*	Pon l 4	~24
	*Portunus trituberculatus*	Por t 4	22
	*Scylla paramamosain*	Scy p 4	20
Troponin C	*Crangon crangon*	Cra c 6	~21
	*Homarus americanus*	Hom a 6	~20
	*Penaeus monodon*	Pen m 6	16.8
Troponin I	*Pontastacus leptodactylus*	Pon l 7	~30
Hemocyanin	*Penaeus monodon*	Pen m 7	76
Triosephosphate isomerase	*Archaeopotamobius sibiriensis*	Arc s 8	~28
	*Crangon crangon*	Cra c 8	~28
	*Penaeus monodon*	Pen m 8	27
	*Procambarus clarkii*	Pro c 8	28
	*Scylla paramamosain*	Scy p 8	28
Filamin C	*Scylla paramamosain*	Scy p 9	90
Cytoplasmic fatty acid-binding protein	*Litopenaeus vannamei*	Lit v 13	15
	*Penaeus monodon*	Pen m 13	20
Glycogen phosphorylase-like protein	*Penaeus monodon*	Pen m 14	95

Note: The allergens were arranged in accordance with their allergen names in the IUIS.

## Data Availability

The original contributions presented in the study are included in the article, further inquiries can be directed to the corresponding author.

## Abbreviations

The following abbreviations are used in this manuscript:

WHO/IUIS	World Health Organization and International Union of Immunological Societies
MW	Molecular weight
PDB	Protein data bank
